# Psychodermatology Profiles in Acne Vulgaris: Quality of Life, Stress, Insomnia, and Depressive Symptoms in a Cross-Sectional Cohort

**DOI:** 10.3390/jcm15166447

**Published:** 2026-08-20

**Authors:** Roxana Manuela Fericean, Ana-Olivia Toma, Iulia Georgiana Bogdan, Horia Silviu Branea

**Affiliations:** 1 Discipline of Dermatology, Faculty of Medicine, “Victor Babes” University of Medicine and Pharmacy, Eftimie Murgu Square 2, 300041 Timisoara, Romania; manuela.fericean@umft.ro (R.M.F.); branea.horia@umft.ro (H.S.B.); 2 Center for the Morphologic Study of the Skin (MORPHODERM), “Victor Babes” University of Medicine and Pharmacy, Eftimie Murgu Square 2, 300041 Timisoara, Romania; 3 Department of Infectious Diseases, Methodological and Research Center for Infectious Diseases (CMCBI), “Victor Babes” University of Medicine and Pharmacy, Eftimie Murgu Square 2, 300041 Timisoara, Romania; 4 Department of Internal Medicine I—Medical Semiotics II, “Victor Babes” University of Medicine and Pharmacy, Eftimie Murgu Square 2, 300041 Timisoara, Romania

**Keywords:** acne vulgaris, quality of life, sleep, depression, stress, psychological

## Abstract

**Background/Objectives**: Acne vulgaris can impair quality of life (QoL) beyond lesion burden, and distress, insomnia, and stress may co-occur. We compared patient-reported burden across treatment-intensity groups used as pragmatic proxies of disease burden and management complexity, quantified associations between clinical severity and psychosocial measures, and modeled predictors of high QoL impairment. **Methods**: Cross-sectional outpatient study (N = 97) in Timișoara, Romania. Severity was assessed using the Global Acne Grading System (GAGS) and scarring grade (0–3). Participants completed Romanian-language versions of the Dermatology Life Quality Index (DLQI), Cardiff Acne Disability Index (CADI), Patient Health Questionnaire-9 (PHQ-9), Insomnia Severity Index (ISI), and Perceived Stress Scale-10 (PSS-10). Group comparisons used analysis of variance (ANOVA)/Kruskal–Wallis and chi-square tests; associations used Spearman correlations. Multivariable ordinary least squares (OLS) regression modeled DLQI; penalized logistic regression modeled DLQI ≥ 11. Treatment intensity was analyzed as a descriptive stratification variable rather than as an independent exposure, and a prespecified sensitivity analysis re-examined the DLQI gradient after adjustment for GAGS and scarring grade. The DLQI ≥ 11 cut-off was prespecified from validated DLQI banding. Clustering, mediation, and network analyses were exploratory and hypothesis-generating in view of the modest sample size. **Results**: DLQI differed across treatment intensity strata (topical only: 11.1 ± 5.7, oral antibiotic + topical: 16.9 ± 7.6, isotretinoin: 20.4 ± 7.1; *p* < 0.001). High impairment (DLQI ≥ 11) occurred in topical only: 40.6%, oral antibiotic + topical: 78.8%, isotretinoin: 87.5% (overall 69.1%; *p* < 0.001). DLQI correlated with PHQ-9 (ρ = 0.59), ISI (ρ = 0.49), and PSS-10 (ρ = 0.60) (all *p* < 0.001). In multivariable analysis, GAGS (B 0.35, *p* < 0.001), PHQ-9 (B 0.27, *p* = 0.017), and PSS-10 (B 0.24, *p* = 0.007) independently predicted DLQI. The combined predictive model achieved an area under the curve (AUC) of 0.841 (5-fold cross-validation, CV) for identifying DLQI ≥ 11. **Conclusions**: In this Romanian outpatient cohort, psychosocial measures explained substantial variability in acne-related disability alongside clinical severity. These cross-sectional findings support brief integrated screening and suggest that higher-burden treatment strata merit particular psychosocial attention, but they should not be interpreted as evidence that treatment intensity itself independently causes worse mental health outcomes. The contribution of this work lies less in confirming known associations than in quantifying, in an under-represented eastern European outpatient setting, how much of the identification of high-impact patients depends on patient-reported rather than lesion-based information.

## 1. Introduction

Acne vulgaris is among the most common inflammatory skin diseases worldwide and is a leading contributor to the nonfatal burden of skin conditions, particularly in adolescents and young adults. Global burden analyses place acne within the highest prevalence of disorders and emphasize its disability burden at the population level, reinforcing that acne should be approached as more than a cosmetic complaint [1,2]. Epidemiologic syntheses estimate that acne affects a substantial fraction of the global population, with meaningful persistence beyond adolescence and a sizable subgroup experiencing chronic or recurrent disease trajectories that extend into early adulthood [3,4]. Recent trend analyses further suggest that the burden in people aged 10–24 years has increased over recent decades across many regions, underscoring the clinical relevance of timely recognition and effective management [5].

Despite the routine use of clinician-rated grading systems to quantify inflammatory and comedonal burden, acne-related disability often reflects domains that are not fully captured by lesion counts alone. The “lived experience” commonly includes embarrassment, concealment and avoidance behaviors, reduced social participation, and anticipatory worry about scarring and long-term appearance changes—dimensions that can persist even when clinical severity is moderate [4]. This mismatch between clinician-rated severity and patient experience motivates the integration of patient-reported outcomes (PROs) into routine acne care, especially for identifying high-impact patients who may require additional supportive interventions [4].

Psychiatric and psychosocial comorbidity is a central component of acne burden. Meta-analytic evidence supports an increased risk of depression and anxiety among individuals with acne, with substantial heterogeneity across settings and populations [6]. Complementing pooled analyses, population-based cohort evidence also indicates elevated depression risk among patients with acne, highlighting that the association is not restricted to highly selected clinical samples [7]. At the individual level, acne-related quality-of-life impairment has been shown to track with symptoms of anxiety and depression, supporting a multidimensional model of disability in which emotional and cognitive responses to appearance can amplify functional impact [8].

Sleep and circadian factors are increasingly recognized as relevant correlates of acne burden and psychosocial distress. Adult studies link greater acne severity to poorer sleep quality, suggesting that sleep disruption may be part of the broader biopsychosocial phenotype in acne [9]. Additional evidence connects acne-related quality-of-life impairment and severity with insomnia and chronotype, implying that circadian preference and sleep disturbance may interact with emotional symptoms and daily functioning [10]. Case–control data also indicate differences in sleep quality, circadian preferences, and mood among patients with acne compared with controls, supporting the clinical rationale for screening sleep symptoms when acne-related distress is prominent [11].

Stress and scarring further contribute to heterogeneity in patient experience. Stress has been associated with acne presence/severity and may exacerbate perceived burden through heightened arousal, rumination, and maladaptive coping behaviors, particularly in high-demand contexts such as medical training [12]. Scarring is often the most enduring “memory” of acne and can produce long-term psychosocial effects; population-based, multi-country data show that facial atrophic scars can impair quality of life (QoL) across cultural settings [13]. Consistent with this, both reviews and empirical studies describe pronounced impacts of acne scarring on self-image and social functioning, with measurable links to depressive symptoms and body-image concerns [14,15].

Although the association between acne and psychosocial burden is well established, three gaps remain that motivated the present work. First, the available evidence is drawn predominantly from western European, North American, and east Asian cohorts, whereas eastern European outpatient populations are markedly under-represented; this matters because access to psychodermatology services, thresholds for systemic therapy, and cultural attitudes to mental health consultation differ substantially across European health systems. Second, psychosocial domains are usually reported in isolation, so that quality of life, depressive symptoms, insomnia, and perceived stress are rarely measured concurrently alongside standardized clinician-rated severity in the same patients; without concurrent measurement the incremental information contributed by each domain cannot be estimated. Third, few studies quantify how much of the identification of high-impact patients is achievable from lesion-based grading alone and how much requires patient-reported information. The present study addresses these gaps by applying a fully Romanian-validated multi-instrument battery in a consecutively sampled outpatient cohort and by formally comparing the discriminative contribution of clinical and psychosocial information. The intended contribution is therefore quantitative and contextual rather than the assertion of a previously unknown association.

This study aimed to quantify how dermatology-specific disability aligns with clinical severity and scarring while capturing key psychodermatology dimensions (stress, insomnia, and depressive symptoms) using Romanian-validated questionnaires. In this Romanian cohort from eastern Europe, we compared patient-reported burden across treatment-intensity groups handled as pragmatic proxies of disease burden, assessed associations among severity/scarring and psychosocial measures, modeled predictors of high QoL impairment, and treated data-driven phenotyping analyses as exploratory tools for characterizing heterogeneous “high-impact” presentations that may benefit from integrated dermatologic and psychosocial care.

## 2. Materials and Methods

### 2.1. Study Design and Setting

This cross-sectional observational study was conducted in outpatient dermatology clinics in Timișoara, Romania. The study was designed to characterize the psychodermatology profile of patients with acne vulgaris by integrating standardized clinician-rated severity measures with validated Romanian-language patient-reported outcome instruments. All participants underwent a structured single-visit assessment consisting of clinical examination, grading of acne severity and scarring, documentation of current treatment intensity, and completion of a questionnaire battery focused on dermatology-specific quality of life, acne-specific disability, depressive symptoms, insomnia, and perceived stress.

### 2.2. Participants and Sampling

Eligible participants were consecutive outpatients aged 16–35 years with clinician-confirmed acne vulgaris and a symptom duration of at least 6 months. Consecutive sampling was used to enhance feasibility and reduce selective recruitment bias in a routine clinical setting. To ensure reliable completion of the self-administered instruments, participants were required to be able to read and understand Romanian and to complete all questionnaire items included in the study battery.

Exclusion criteria were the presence of another inflammatory dermatosis considered by the evaluating clinician to be the principal cause of current quality-of-life impairment, current systemic corticosteroid therapy, and acute psychiatric instability requiring urgent intervention or preventing valid questionnaire completion. A pragmatic target sample of approximately 100 patients was set in order to support subgroup comparisons and multivariable modeling within the constraints of a single-region outpatient study. Ninety-seven participants completed the full assessment and were included in the final analytic cohort.

At the time of assessment, patients were categorized into three treatment intensity strata reflecting the current therapeutic approach: topical-only therapy, oral antibiotic plus topical therapy, and systemic isotretinoin therapy. These strata were used as clinically interpretable proxies of overall treatment intensity and disease management complexity; however, throughout the analysis they were interpreted as pragmatic markers of underlying disease burden rather than as independent causal exposures, because treatment allocation in acne is inherently shaped by baseline severity, scarring, prior treatment failure, and clinician judgment. The grouping strategy allowed comparison of clinician-rated severity and psychodermatology burden across progressively more intensive therapeutic categories. Because allocation to these strata is driven by the same clinical features that also drive patient-reported burden, treatment intensity was deliberately not entered into the multivariable models as an independent predictor of quality of life; it is used throughout as a descriptive stratification variable only.

### 2.3. Clinical Assessment and Questionnaires

Clinical acne severity was assessed by the treating/evaluating dermatologist using the Global Acne Grading System (GAGS), a standardized instrument that captures regional involvement and lesion burden to generate an overall severity score. In parallel, acne scarring was graded on a simple ordinal scale from 0 to 3, where 0 indicated no apparent scarring, 1 mild scarring, 2 moderate scarring, and 3 severe scarring. For subgroup analyses, high scarring was defined a priori as a scarring grade of 2 or higher.

Participants then completed a Romanian-language questionnaire battery composed of validated, brief instruments chosen for clinical feasibility and psychometric robustness. Dermatology-specific quality of life was measured using the Dermatology Life Quality Index (DLQI), with higher scores indicating greater impairment in day-to-day functioning attributable to skin disease. Acne-specific disability was assessed with the Cardiff Acne Disability Index (CADI), which captures the impact of acne on emotions, social interactions, and self-perception.

Depressive symptoms were measured with the Patient Health Questionnaire-9 (PHQ-9), insomnia severity with the Insomnia Severity Index (ISI), and perceived stress with the Perceived Stress Scale-10 (PSS-10). For all instruments, total scores were calculated according to standard scoring procedures, and higher values indicated greater symptom burden or worse quality of life. Questionnaire totals were analyzed primarily as continuous variables, while selected thresholds were additionally used to define clinically interpretable binary endpoints. Specifically, high QoL impairment was defined as DLQI ≥ 11, and moderate-to-severe depressive symptom burden was defined as PHQ-9 ≥ 10.

Demographic and clinical covariates recorded on the standardized case-report form at the study visit comprised age, sex, area of residence, body mass index, acne duration, GAGS score, scarring grade, and current treatment stratum. The anatomical distribution of lesions, the number and nature of previous treatment failures, and previous or current psychiatric treatment were not systematically documented on that form. These variables were therefore unavailable for adjustment and are treated as unmeasured confounders rather than as modeled covariates; their absence is addressed explicitly in the limitations section.

### 2.4. Outcomes and Definitions

The primary outcome of the study was acne-related quality-of-life burden as measured by total DLQI score, analyzed both as a continuous outcome and as a binary clinically meaningful endpoint (DLQI ≥ 11). This threshold was prespecified in the study protocol before any analysis was undertaken and is consistent with the validated interpretive banding of the DLQI proposed by Hongbo et al., in which scores of 0–1 indicate no effect, 2–5 a small effect, 6–10 a moderate effect, 11–20 a very large effect, and 21–30 an extremely large effect on a patient’s life [16]. Dichotomizing at 11 therefore reproduces an existing, externally validated band boundary rather than introducing a new one: it separates patients in whom skin disease has at most a moderate effect (0–10) from those in whom the effect is very large or extremely large (11–30). No alternative cut-off points were examined and the threshold was not derived from the present data, which avoids data-driven cut-off point selection and the optimism it introduces. The resulting binary endpoint identifies participants with substantial dermatology-specific impairment who may require more intensive supportive management in addition to acne-directed treatment.

Secondary outcomes included acne-specific disability (CADI total), depressive symptom burden (PHQ-9 total), insomnia severity (ISI total), and perceived stress (PSS-10 total). The main clinical exposures of interest were GAGS severity score, scarring grade, and treatment-intensity group. Additional demographic and clinical characteristics, including age, sex, body mass index, and acne duration, were summarized descriptively and incorporated into selected models as covariates where appropriate.

To explore the possibility that psychosocial burden modified the relationship between depression and quality-of-life impairment, insomnia severity was further examined as an effect-modifying domain. ISI scores were stratified into tertiles for exploratory subgroup evaluation, and interaction terms involving PHQ-9 x ISI were examined in model-building steps. Beyond hypothesis-driven modeling, the study also explored whether patients clustered into distinct psychodermatology phenotypes when severity and psychosocial measures were considered jointly; these latter analyses were considered hypothesis-generating rather than confirmatory.

### 2.5. Statistical Analysis

Continuous variables are presented as mean ± standard deviation (SD) (one-decimal precision); categorical variables as n (%). Group comparisons used one-way analysis of variance (ANOVA) when appropriate and Kruskal–Wallis otherwise; categorical variables used chi-square tests. Two-group comparisons (high vs. low scarring) used Welch’s *t*-tests and chi-square tests. Associations used Spearman correlations. Multivariable ordinary least squares (OLS) regression modeled DLQI. Penalized logistic regression (L2) modeled DLQI ≥ 11 with bootstrap confidence intervals. K-means clustering (standardized inputs)-derived phenotypes and mediation used nonparametric bootstrap resampling. Model discrimination was assessed via 5-fold cross-validated area under the receiver operating characteristic (ROC) curve (AUC). Given the modest sample size, the penalized regression, clustering, mediation, network, and discrimination analyses were interpreted as exploratory and hypothesis-generating complements to the primary descriptive and multivariable analyses, not as stand-alone proof of stable latent structure or causal pathways.

Analyses were organized into a prespecified hierarchy in order to keep the primary clinical message distinct from secondary methodological exploration. The primary analyses were the descriptive comparison of clinical and patient-reported measures across treatment intensity strata and scarring categories, the Spearman correlation structure, and the multivariable OLS model of DLQI. All remaining procedures—penalized logistic regression, k-means clustering, serial mediation, Graphical Lasso network estimation, and cross-validated discrimination—were designated secondary and exploratory in advance and are reported as hypothesis-generating only. No formal adjustment for multiple comparisons was applied; *p* values arising from secondary analyses are therefore interpreted descriptively rather than as confirmatory tests, and no estimate from these analyses is used to support an inference of causality.

Confounding was addressed in three ways. First, covariate balance across treatment intensity strata was examined for age, sex, area of residence, body mass index, acne duration, and scarring grade. Second, the multivariable OLS model of DLQI and the penalized logistic model of DLQI ≥ 11 were adjusted a priori for age, sex, GAGS score, and scarring grade in addition to the psychosocial predictors, so that the reported psychosocial coefficients are conditional on both demographic characteristics and clinician-rated disease severity. In order to limit the number of estimated parameters relative to the available sample size, acne duration was evaluated as a candidate covariate but did not differ across strata and was not retained. Third, because allocation to treatment intensity strata is itself determined by severity, a prespecified sensitivity analysis re-examined the difference in DLQI across strata after adjustment for GAGS score and scarring grade using analysis of covariance. This was undertaken in order to establish whether the observed gradient is attributable to underlying disease severity rather than to treatment category as such. Anatomical lesion distribution, previous treatment failures, and previous psychiatric treatment were not captured on the case-report form and could not be adjusted for.

The exploratory procedures were deliberately constrained by the available sample, and the specific constraints are stated here so that readers can judge their stability. With 97 participants and 67 events for the DLQI ≥ 11 endpoint, the logistic model was restricted to six predictors, corresponding to approximately 11 events per variable, and L2 penalization with bootstrap confidence intervals (2000 resamples) was used to reduce optimism and to stabilize coefficients in the presence of correlated predictors and near-separation. The number of clusters was fixed at three on the basis of the silhouette criterion together with clinical interpretability, rather than selected post hoc to maximize between-cluster separation; formal cluster stability was not established. Mediation estimates were obtained by nonparametric bootstrap because the sampling distribution of an indirect effect is asymmetric, although bootstrap resampling cannot remedy the absence of temporal ordering inherent to a cross-sectional design, and the mediation model should therefore be read as a description of statistical association compatible with several causal orderings rather than as a test of one. Network edges were estimated with Graphical Lasso regularization, which shrinks weak partial correlations toward zero and limits spurious edges, though edge weights derived from a sample of this size carry wide uncertainty that is not quantified here. Discrimination was assessed by 5-fold cross-validation, which provides internal validation only; no external or temporal validation was performed, and the reported AUC values should not be interpreted as expected performance in an independent population. These constraints are the reason the corresponding results are presented throughout as provisional and in need of replication in larger cohorts.

## 3. Results

The analytic cohort included 97 acne outpatients across three treatment intensity strata. Higher clinical severity and scarring burden aligned with worse dermatology-specific disability and higher psychosocial symptom burden across analyses. Table 1 shows a coherent severity gradient across treatment intensity strata. GAGS increases stepwise and is mirrored by higher DLQI/CADI, indicating rising disability with clinical burden. Psychosocial measures (PHQ-9, ISI, PSS-10) also tend to increase with treatment intensity, consistent with broader strain in more severe or scarring-prone acne. Because treatment strata in this outpatient sample reflect underlying therapeutic selection, these gradients should be interpreted descriptively as burden gradients rather than as evidence that treatment intensity itself independently worsened psychosocial outcomes.

**Figure 1:**
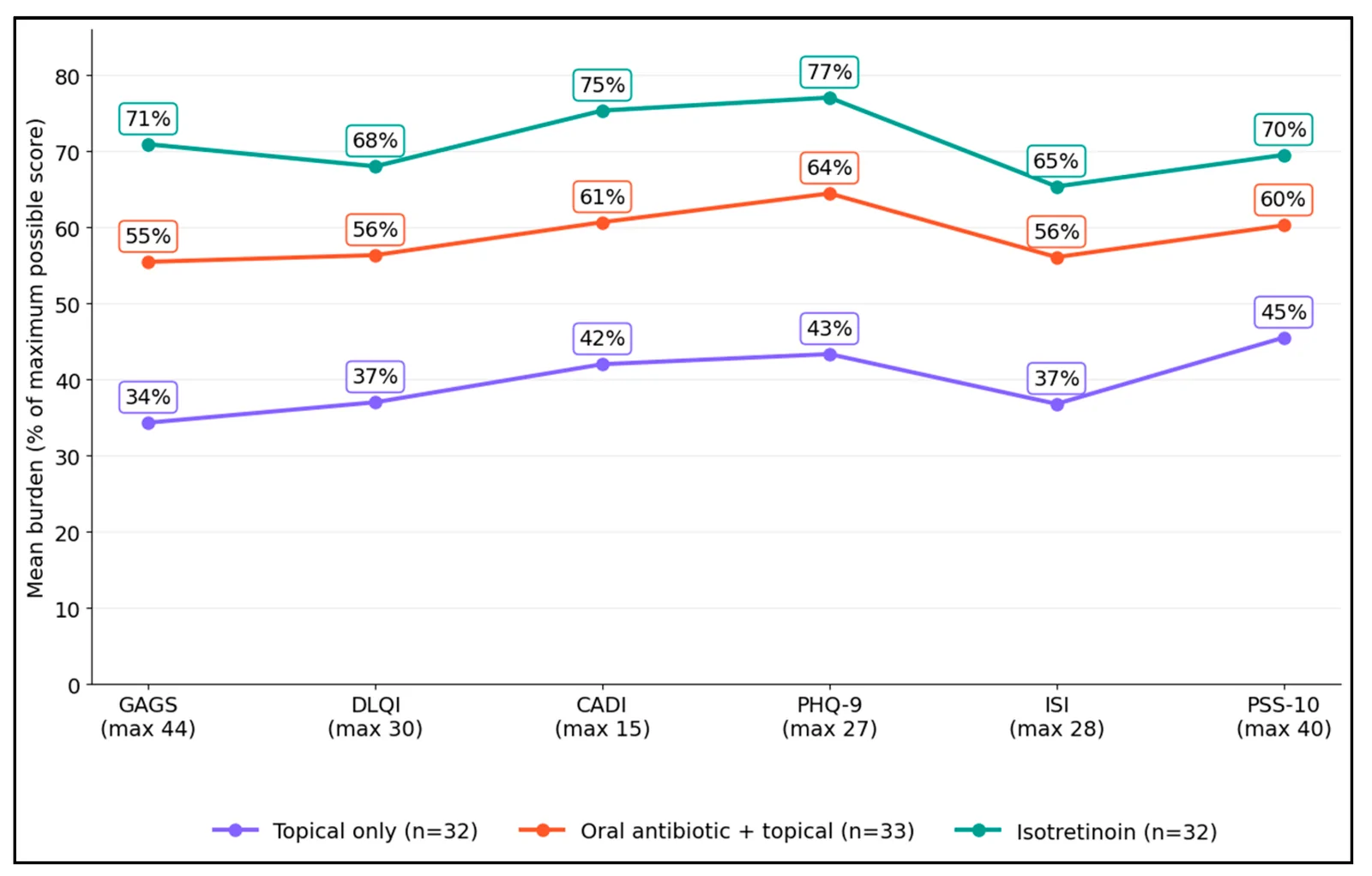
Integrated psychodermatology burden profile across treatment intensity groups.

Patients receiving isotretinoin showed the highest relative burden across all domains, followed by those receiving oral antibiotic plus topical therapy, while the topical-only group had the lowest values. The gradient was consistent not only for clinical severity (GAGS), but also for dermatology-specific quality-of-life impairment (DLQI), acne-specific disability (CADI), depressive symptoms (PHQ-9), insomnia severity (ISI), and perceived stress (PSS-10), as presented in Figure 1. This pattern is compatible with confounding by indication, whereby patients receiving systemic therapy represent the subgroup with the greatest baseline disease burden and management complexity.

The three treatment intensity strata were closely comparable with respect to age, sex, area of residence, body mass index, and acne duration (all *p* > 0.25; Table 1), but differed substantially in clinician-rated disease severity. Mean GAGS score rose from 15.1 +/− 3.8 in the topical-only stratum to 24.4 +/− 4.6 in the oral antibiotic plus topical stratum and 31.2 +/− 5.4 in the isotretinoin stratum (*p* < 0.001), a difference of more than two standard deviations between the extreme groups. The proportion of patients with high scarring also increased across strata (31.2%, 48.5%, and 59.4%), but this gradient did not reach the conventional significance threshold (*p* = 0.075). Because severity differed markedly across strata while demographic and disease duration characteristics did not, the DLQI gradient shown in Table 1 cannot be attributed to treatment category independently of disease severity; the severity-adjusted sensitivity analysis addressing this question directly is presented at the end of the Results Section.

Table 2 isolates scarring burden. High scarring (grade ≥ 2) is associated with higher severity and materially worse QoL and psychosocial outcomes, supporting routine distress screening in scarring-prone patients.

Table 3 highlights an integrated burden structure: DLQI correlates strongly with depression, stress, and insomnia, while clinical severity and scarring correlate more modestly, supporting multivariable modeling.

Table 4 indicates that clinical severity (GAGS) remained independently associated with disability, whereas the apparent effect of scarring was attenuated after adjustment. PHQ-9 and PSS-10 contributed strongly beyond clinical phenotype, supporting person-centered screening.

Table 5 models a clinically interpretable endpoint (DLQI ≥ 11) using penalized logistic regression with bootstrap CIs. Clinical severity and psychosocial measures both contributed to risk, although the model should be interpreted cautiously because of the modest sample size and the cross-sectional design.

Table 6 presents exploratory data-driven phenotypes combining severity and psychosocial burden, suggesting heterogeneous needs beyond conventional severity categories.

Table 7 presents exploratory mediation estimates for a pathway in which scarring relates to higher stress and depressive symptoms, which in turn may contribute to higher disability. Bootstrap indirect effects are reported as hypothesis-generating rather than causal.

Table 8 shows acceptable-to-excellent internal consistency across instruments, supporting coherent total scores in this sample.

Table 9 presents exploratory conditional links from a regularized network; DLQI connects to both psychosocial and clinical factors, reinforcing a multifactorial disability construct while warranting replication in larger datasets.

Table 10 shows that the psychosocial and combined models discriminated DLQI ≥ 11 better than the clinical-only model in 5-fold cross-validation.

**Figure 2:**
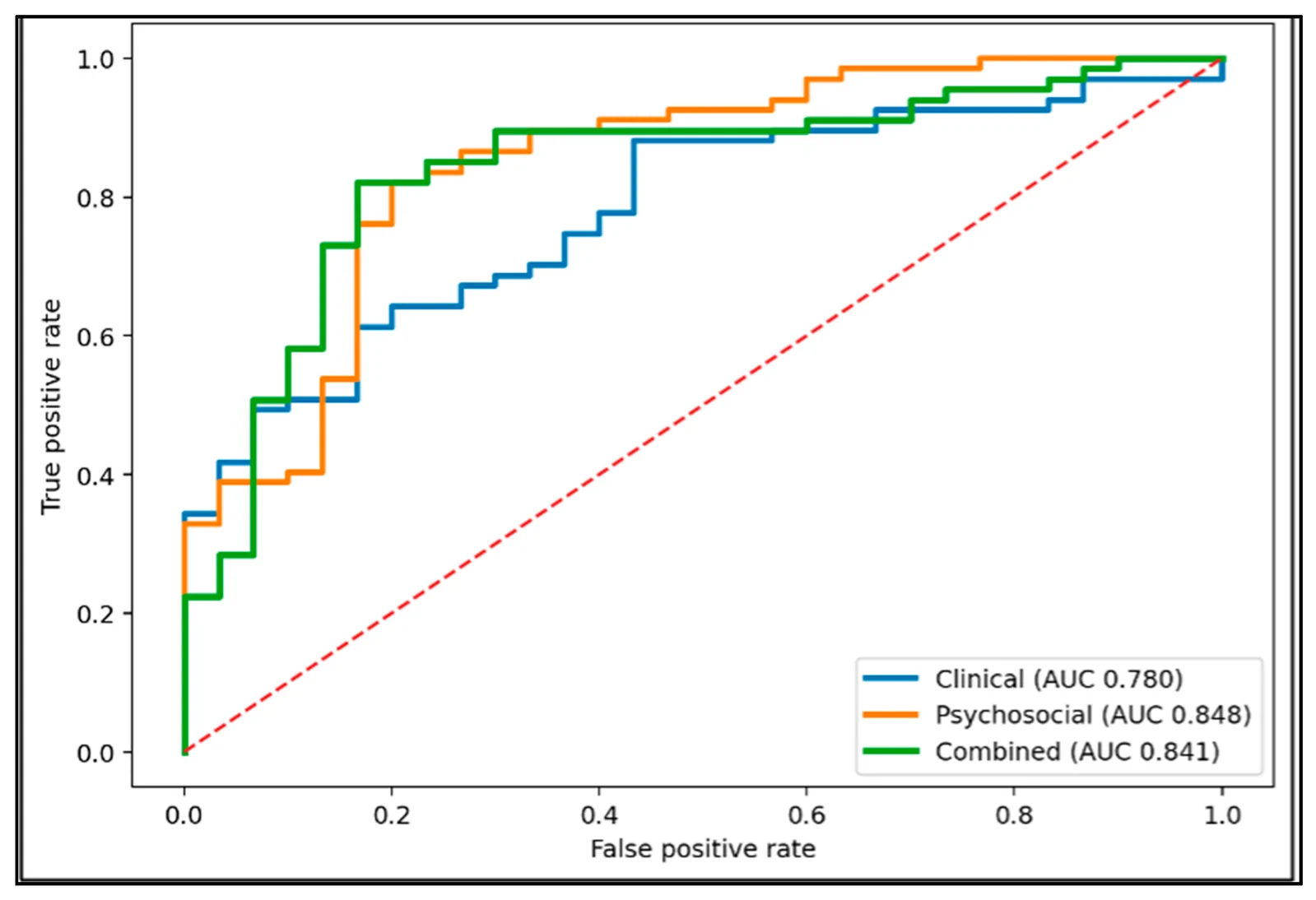
ROC curves for predicting high QoL impairment (DLQI ≥ 11) using 5-fold cross-validated models.

Figure 2 compares discrimination for DLQI ≥ 11. The combined model achieved AUC 0.841, outperforming the clinical-only model (AUC 0.780) and performing similarly to the psychosocial-only model (AUC 0.848).

To determine whether the difference in dermatology-specific quality of life across treatment intensity strata reflected treatment category itself or the underlying disease severity that governs treatment allocation, the prespecified sensitivity analysis summarized in Table 11 compared unadjusted group means with means adjusted successively for GAGS score, for GAGS score and scarring grade, and for GAGS score, scarring grade, age, and sex. This analysis is reported to allow readers to judge directly how much of the apparent treatment intensity gradient survives adjustment for clinical severity, and it should be read together with the multivariable model in Table 4, in which GAGS remained independently associated with DLQI while treatment stratum was, by design, not entered as a predictor.

## 4. Discussion

### 4.1. Principal Findings and Interpretation

The high prevalence of clinically meaningful QoL impairment (DLQI ≥ 11) and the steep gradient across treatment intensity strata indicate that patient-perceived burden in acne is associated with both objective disease activity and broader psychosocial load, and is not captured by lesion counts alone. Because all measurements were obtained at a single visit, the direction of these associations cannot be established, and the interpretations offered throughout the below distinguish between what the data statistically support and what is biologically plausible but untested. Crucially, the treatment intensity gradient should not be read as an exposure effect. In routine acne care, patients receiving oral antibiotics or isotretinoin are typically those with greater baseline severity, scarring burden, prior treatment failure, or referral complexity. The present strata are therefore best understood as pragmatic severity management categories, and the worse patient-reported outcome profile in the isotretinoin group is most plausibly explained by confounding by indication rather than harm attributable to treatment itself. Population-based evidence supports acne as a condition with a measurable downstream mental health risk at scale, including increased depression risk in real-world settings, underscoring the clinical relevance of routine distress detection even outside psychiatry-focused samples [7]. Earlier dermatology QoL work also demonstrates that acne can produce impairment comparable to other chronic dermatoses in adult practice, reinforcing the need to treat QoL as a co-primary target rather than a secondary “soft” endpoint [17]. Multi-country adult acne data further highlight that psychosocial impact is common and clinically non-trivial across diverse European contexts, aligning with the observed heterogeneity in disability that cannot be inferred reliably from severity grading alone [18].

The mean PHQ-9 score of 20.8 in the isotretinoin stratum falls within the severe depressive symptom range and deserves explicit clinical attention. In a cross-sectional outpatient cohort such as ours, this finding likely reflects selection and referral mechanisms that concentrate patients with more severe, chronic, scarring, and psychosocially disruptive acne into the most intensive treatment category. In other words, the value is clinically alarming and should trigger screening awareness, but it should not be over-interpreted as evidence of an adverse mood effect of isotretinoin in this dataset. The strong correlations between DLQI and depressive symptoms/stress, together with the independent contributions of PHQ-9 and PSS-10 in multivariable models, are consistent with the literature showing close coupling between acne-related disability and affective burden. Prior clinical studies report that acne-specific and dermatology-specific QoL measures are associated with both anxiety and depression symptomatology, and that psychiatric symptom burden may remain prominent even when clinician-rated severity is not extreme, supporting integrated assessment rather than “severity-only” decision-making [19]. In addition, acne cohorts in European settings have reported clinically relevant rates of depressive symptoms and suicidal ideation alongside QoL impairment, emphasizing the importance of structured screening pathways and clear escalation plans when high-risk signals emerge on brief questionnaires [20].

The relationship between stress, sleep, and mood remained clinically meaningful in our cohort, but the adjusted models also showed an important null result: ISI was not independently significant in either the OLS or penalized logistic regression models once PHQ-9 and PSS-10 were entered. This pattern suggests that insomnia may travel with broader distress and stress rather than contribute substantial independent incremental information after shared variance is considered. Clinically, sleep symptoms still matter, but our data support presenting insomnia as part of the psychodermatology burden profile rather than overstating it as an autonomous predictor in this sample. These findings are concordant with broader inflammatory skin disease evidence linking perceived stress to dermatologic burden at the population level. Large-scale analyses that include acne among other inflammatory dermatoses show that perceived stress is frequent and clinically salient, supporting the plausibility that stress acts as both a correlate of visible disease and a contributor to the lived burden of symptoms and social functioning [21]. Context-specific stressors may further modulate acne outcomes; observational data in medical students suggest academic stress can be associated with acne dynamics, providing a pragmatic lens for interpreting elevated PSS-10/PHQ-9 scores in younger outpatient cohorts [22]. Sleep-related factors are increasingly implicated: associations among acne severity, insomnia, and chronotype have been reported, and case–control data link acne with poorer sleep quality and mood differences, consistent with the clinical utility of brief insomnia screening when QoL impairment appears disproportionate to clinical findings [23,24].

Scarring also warrants a more restrained interpretation than a binary significant/non-significant reading would suggest. The comparison for high QoL impairment (DLQI ≥ 11) between high- and low-scarring groups narrowly missed the conventional threshold (80.0% vs. 59.6%, *p* = 0.052). We interpret this as a clinically suggestive signal constrained by sample size rather than as evidence of no association, especially because scarring was found to be associated with worse continuous PRO scores and heavier depression/stress burden. Scarring-associated differences in PROs and psychosocial scores, alongside indirect effects consistent with stress/depression pathways, are compatible with the hypothesis that scarring acts as a “visibility amplifier” capable of sustaining distress even when active inflammation fluctuates. We emphasize that this is an interpretive hypothesis rather than a finding: the mediation estimates reported in Table 7 describe a pattern of statistical association within a single cross-sectional dataset, and the same correlation structure would be produced by several alternative orderings, including one in which pre-existing distress increases manipulation of lesions and thereby scarring, or one in which an unmeasured third factor drives both. Confirming the proposed ordering would require longitudinal measurement of scarring, stress, and mood. Studies focused specifically on acne scars demonstrate meaningful associations among scarring, depression, body image disturbance, and QoL impairment, which is consistent with, though not confirmatory of, the interpretation that scarring burden contributes to persistent psychosocial disability and may warrant proactive counseling and expectation management [25]. Contemporary reviews emphasize that acne scarring can represent a distinct psychosocial driver—partly overlapping with active acne, yet also extending beyond it through stigma-related stress, regret/self-blame, and avoidant behaviors—providing a candidate interpretive framework for the observed mediation patterns involving stress and depressive symptoms, which our design can describe but cannot test [13]. Health utility work further indicates that scarring can impose measurable quality-of-life penalties and influences patient valuations of improvement, reinforcing early prevention strategies and structured shared decision-making around scar-minimizing treatment plans [26]. Consistent with this interpretation, studies of patients treated with oral isotretinoin have reported improvements in anxiety, depressive symptoms, and quality of life during therapy, supporting the view that the high psychosocial burden observed in the isotretinoin stratum reflects baseline disease severity rather than an adverse effect of treatment itself [27,28].

### 4.2. Broader Psychosocial and Environmental Determinants of Acne Burden

The instruments used here capture the emotional and symptomatic dimensions of acne burden, but they do not exhaust its determinants. Acne-related distress is generated at the interface between a visible lesion and a social environment, and several of the pathways involved were not quantified in this study. Stigma and anticipated negative evaluation are among the most consistently described: patients with visible facial disease report staring, unsolicited advice, and attributions of poor hygiene or self-neglect, and large European multicenter data indicate that clinically relevant depressive and anxiety symptoms cluster in dermatology outpatients to a degree that lesion severity alone does not predict [29]. Body image disturbance offers a related mechanism, one in which appearance becomes disproportionately central to self-evaluation, so that objectively modest lesion counts can sustain marked distress; this may partly explain the heterogeneity we observed in DLQI at any given GAGS score.

Social functioning and interpersonal relationships are affected in parallel, through concealment behaviors, avoidance of photography and video calls, reduced participation in dating and peer activities, and withdrawal from settings involving close visual contact. Educational and occupational pressures add a further layer that is particularly relevant to the age range studied here, as 16–35 years spans examinations, university entry, job interviews, and early career development, all of which involve appearance-sensitive evaluation; the reported association between academic stress and acne dynamics in student populations is consistent with this reading [22]. Family support and socioeconomic context modulate these effects in both directions. Supportive families and adequate financial resources facilitate early consultation, adherence to treatment, and access to scar-directed procedures that are frequently not reimbursed, whereas financial constraint, limited insurance coverage, or the dismissal of acne as a trivial adolescent complaint delay care and may prolong exposure to active disease and to scarring. In Romania, where dedicated psychodermatology services are not widely available and where mental health consultation continues to carry stigma, these contextual factors are likely to be especially salient.

None of these domains were measured in the present study, and their contribution therefore cannot be estimated from our data. They are raised for two reasons. First, a purely symptom-based account of acne burden is incomplete, and readers should not infer from the variance explained by PHQ-9, ISI, and PSS-10 that these instruments capture the whole of the psychosocial picture. Second, a biopsychosocial framing identifies intervention targets that lie outside the prescription itself—stigma reduction, body image work, engagement with family and educational settings, and improved access to affordable scar-directed care—and these are the levers most likely to be available to clinicians when disease severity has already been treated adequately. Future work in this cohort should incorporate validated measures of stigmatization, body image, social appearance anxiety, and socioeconomic position alongside the present battery.

### 4.3. Insomnia as a Clinically Meaningful Component of Burden

The position of insomnia within this profile deserves comment that is neither dismissive nor overstated. ISI scores rose across treatment strata and were substantially higher in patients with high scarring, yet insomnia did not retain independent significance once depressive symptoms and perceived stress were entered into the models. It would be a mistake to read this as evidence that sleep is unimportant. Evidence from other clinical populations makes clear that insomnia carries its own burden largely irrespective of the condition it accompanies: in a large survey of Italian night-shift nurses, insomnia was present in approximately two-thirds of respondents and was framed explicitly as a determinant of impaired quality of life, psychological distress, and reduced daily functioning rather than as an incidental symptom [30]. That population differs from ours in nearly every respect—occupational rather than dermatological, older, and exposed to a circadian stressor rather than to a visible skin disease—and the comparison is instructive precisely for that reason, as it indicates that the functional consequences of disturbed sleep are relatively independent of the disorder that produces it. Applied to acne, this suggests that the statistical redundancy of ISI in our adjusted models reflects shared variance with stress and mood in this particular sample rather than clinical irrelevance. Sleep loss plausibly interacts with acne burden in both directions, through daytime fatigue and irritability that erode coping capacity, and through evening rumination about appearance that delays sleep onset. Insomnia is also directly actionable: brief behavioral and sleep hygiene interventions are inexpensive, carry little risk, and do not require psychiatric referral. On this basis we continue to recommend routine assessment of insomnia alongside depressive symptoms and stress in patients with acne, while presenting it as one component of an integrated burden profile rather than as an autonomous predictor of disability.

### 4.4. Implications for Integrated and Multidisciplinary Psychodermatology Care

These observations have practical consequences for how acne services are organized. Both the psychosocial-only model (AUC 0.848) and the combined model (AUC 0.841) discriminated high impairment better than the clinical-only model (AUC 0.780) in internal validation, which indicates that severity grading alone is an incomplete basis for identifying patients who need more than a prescription. The practical implication is that brief validated instruments should be embedded in routine acne consultations rather than reserved for patients who appear obviously distressed. The battery used here is feasible in ordinary practice: DLQI, PHQ-9, ISI, and PSS-10 can be completed in the waiting room in under ten minutes, scored immediately, and repeated at follow-up, and all instruments showed acceptable-to-excellent internal consistency in this sample (Table 8).

Routine measurement with patient-reported outcome measures supports care in three concrete ways. It enables earlier psychological intervention, as patients frequently do not volunteer low mood, sleep disruption, or social withdrawal during a consultation framed around lesion counts, and a completed questionnaire provides a low-threshold entry point to a conversation that might otherwise never occur. It supports shared decision-making, because a documented DLQI in the very-large-effect band is a legitimate and discussable input into decisions about systemic therapy or scar-directed procedures; reviewing scores together with the patient makes the reasoning behind treatment escalation explicit rather than opaque, and gives patients whose lesion counts are modest but whose disability is high a language in which to advocate for themselves. It also structures collaboration between dermatologists, psychologists, and mental health professionals, because an agreed numerical threshold—for example PHQ-9 ≥ 10 with functional impairment, or any positive response to the PHQ-9 self-harm item—converts referral from a matter of individual clinical impression into an auditable pathway with defined responsibilities on each side.

This is the direction in which the field is moving. The European position paper on psychodermatology argues for psychosocial assessment to be embedded within routine dermatological care rather than confined to a small number of specialist centers and identifies the shortage of trained personnel and of structured referral pathways as the principal barriers to doing so [31]. Integrated dermatology–psychiatry service models developed elsewhere provide practical templates, including joint clinics, consultation–liaison arrangements, and stepped-care approaches in which the intensity of psychological input is matched to measured need [32]. Implementing such a model in settings comparable to ours requires modest rather than substantial investment: agreement on which instruments to administer, a documented threshold for escalation, and an identified referral partner willing to accept dermatology referrals. Whether such screening improves patient outcomes rather than merely detecting distress remains an open question that our cross-sectional design cannot address, and prospective evaluation of screening-plus-referral pathways, with quality of life and depressive symptoms as outcomes, is the logical next step.

### 4.5. Novel Contribution and Relationship to the Existing Literature

It is important to state plainly what this study does and does not add. That acne is associated with depressive symptoms, anxiety, impaired quality of life, and sleep disturbance is well established, and we do not present these associations as new. The contribution is more specific and rests on four points. First, the cohort is Romanian and eastern European, a region substantially under-represented in the psychodermatology literature; the multi-country European studies that do exist [18,29] include few or no Romanian centers, so the magnitude of burden in this health system context has largely been assumed by extrapolation rather than measured directly. Second, five domains—dermatology-specific quality of life, acne-specific disability, depressive symptoms, insomnia, and perceived stress—were measured concurrently with standardized clinician-rated severity and scarring in the same patients using fully Romanian-validated instruments, whereas most prior work examines one or two psychosocial domains at a time and therefore cannot estimate the unique information each contributes once the others are accounted for.

Third, the head-to-head comparison of clinical, psychosocial, and combined models quantifies something that is usually asserted qualitatively: how much of the identification of high-impact patients is achievable from lesion-based grading alone, and how much requires patient-reported information. The observation that a psychosocial-only model discriminated at least as well as one containing clinician-rated severity is, to our knowledge, not something that has previously been quantified in an acne cohort, and it carries the clearest implication for practice of anything reported here. Fourth, we report a negative result that is easily lost in this literature: insomnia, despite strong bivariate associations with every other measure, did not contribute independently after adjustment. Reporting this transparently rather than emphasizing only the significant associations is itself a modest contribution to a field in which selective reporting of positive psychosocial correlations is common.

Set against existing work, our estimates of quality-of-life impairment and depressive symptom burden lie at the higher end of published ranges. Reported rates of clinically relevant depressive symptoms in acne populations vary widely with setting and instrument, and our figures most plausibly reflect a referral-based outpatient sample with substantial systemic therapy use rather than a community population; they should not be read as prevalence estimates for acne in the general Romanian population, and direct comparison with community-based studies would be misleading. Conversely, the relative ordering we observe—psychosocial measures tracking disability more closely than lesion counts—is consistent across the published literature and appears robust to setting. Distinguishing these two levels of comparison, absolute magnitude versus relative structure, is necessary when placing regional cohort data alongside multinational studies.

The discrimination analyses should likewise be read as proof of concept rather than as a finalized clinical prediction tool. In our sample, the combined model (AUC 0.841) improved on the clinical-only model (AUC 0.780) but performed similarly to the psychosocial-only model (AUC 0.848), suggesting that brief psychosocial measures carry much of the signal for high QoL impairment. The main contribution of the present study is therefore not to claim entirely novel associations, but to show that this multidimensional acne burden is clearly present in a Romanian outpatient cohort from eastern Europe, a setting that is less represented in the psychodermatology literature than large western European or multinational samples. Taken together, these findings support implementation of a brief, standardized psychodermatology battery as part of routine acne visits, with predefined thresholds for counseling, sleep/stress intervention, or mental health referral when high-impact profiles are detected. This clinical implication should still be viewed through the lens of cautious interpretation and future external validation. It is worth separating explicitly what these data support from what they merely suggest. The data support the following: quality-of-life impairment is common in this cohort; DLQI covaries strongly with depressive symptoms, perceived stress, and insomnia; GAGS, PHQ-9, and PSS-10 are each independently associated with DLQI after mutual adjustment; and a model containing only patient-reported measures discriminated high impairment at least as well as one containing clinician-rated severity. The following are plausible but untested in this dataset: that stress and depressive symptoms lie on a causal pathway between scarring and disability; that the three clusters correspond to reproducible patient phenotypes rather than to arbitrary partitions of a continuous distribution; that the network edges represent stable conditional dependencies; and that screening would improve outcomes rather than simply identify distress. Each of these belongs to the category of hypotheses generated for prospective testing, not conclusions established here.

### 4.6. Study Limitations

Several limitations warrant explicit emphasis. First, the study was cross-sectional, so temporal ordering and causality cannot be inferred. Second, treatment intensity strata were observational and strongly confounded by indication; patients receiving isotretinoin or oral antibiotics were, by clinical logic, those with greater baseline disease burden, which prevents interpretation of treatment category as an independent exposure. Third, the sample size was modest relative to the breadth of the statistical toolkit, so the clustering, mediation, network, and discrimination analyses should be viewed as exploratory and hypothesis-generating rather than definitive demonstrations of stable latent structure, causal pathways, or transportable prediction performance. Fourth, several potentially relevant confounders were not captured on the standardized case report form and could therefore not be adjusted for. Three deserve specific mention because they were raised in review and because each could plausibly bias the reported estimates. The anatomical distribution and visibility of lesions was not recorded, although facial involvement would be expected to affect quality-of-life impairment independently of total lesion burden, so that two patients with identical GAGS scores may differ substantially in disability. The number and nature of previous treatment failures was not documented, although this influences both current treatment allocation and patient expectations, and is likely to contribute to the treatment intensity gradient we observed. Previous or current psychiatric treatment was not ascertained, which is a material omission given that antidepressant or anxiolytic therapy would be expected to affect PHQ-9 and PSS-10 responses directly, and that patients already in psychiatric care may differ systematically in help-seeking behavior. Anxiety symptoms, socioeconomic position, educational and occupational demands, family support, hormonal contributors such as polycystic ovary syndrome or hormonal contraception, and treatment adherence were likewise unmeasured. Residual confounding from these sources cannot be excluded, and the adjusted estimates presented here should be read as partially rather than fully adjusted; prospective studies in this area should record these variables at baseline. Fifth, all questionnaires were self-reported and completed at a single sitting, so common-method variance is likely to have inflated the observed inter-correlations to some degree; the magnitude of the correlations among PHQ-9, ISI, and PSS-10 should be read with this in mind. Sixth, severity grading and scarring assessment were performed by the treating clinician without independent duplicate assessment, so inter-rater reliability could not be quantified. Finally, the cohort was drawn from a single Romanian region, which strengthens contextual relevance but limits broad generalizability; replication in larger, multicenter eastern European cohorts with external validation is needed.

These limitations do not negate the clinical message of the study, but they do narrow its appropriate interpretation: the manuscript is strongest as a carefully described regional psychodermatology profile with clinically useful screening signals, not as evidence of causation or as a definitive predictive modeling paper.

## 5. Conclusions

In this Romanian outpatient acne cohort, psychosocial measures were closely linked to dermatology-specific disability and added clinically useful context beyond severity and scarring. Because the design was cross-sectional and treatment intensity functioned as a proxy for underlying disease burden, the findings should be interpreted as associative rather than causal. Brief validated questionnaires may nevertheless help identify high-impact profiles and support more integrated acne care. The specific contribution of this work is to quantify that relationship in an eastern European outpatient setting that is poorly represented in the existing literature, and to show that a model built solely from brief patient-reported measures identified high-impact patients at least as well as one built from clinician-rated severity. The exploratory phenotyping, mediation, and network analyses should be read as generating hypotheses for prospective testing rather than as establishing mechanistic pathways, and prospective multicenter studies recording lesion distribution, treatment history, and psychiatric history are required before any of these observations can be regarded as established.

## Figures and Tables

**Table 1:** Baseline characteristics, clinical severity, and patient-reported outcomes by treatment-intensity group (N = 97).

Characteristic	Topical Only (n = 32)	Oral Antibiotic + Topical (n = 33)	Isotretinoin (n = 32)	*p* Value
Age, years	22.4 ± 4.1	22.6 ± 4.6	23.2 ± 4.1	0.725
Body mass index, kg/m^2^	23.6 ± 2.7	24.1 ± 2.1	23.3 ± 2.4	0.575
Acne duration, years	3.1 ± 2.4	2.9 ± 2.1	2.9 ± 2.1	0.993
GAGS severity score	15.1 ± 3.8	24.4 ± 4.6	31.2 ± 5.4	<0.001
DLQI total (0–30)	11.1 ± 5.7	16.9 ± 7.6	20.4 ± 7.1	<0.001
CADI total (0–15)	6.3 ± 2.6	9.1 ± 3.2	11.3 ± 3.2	<0.001
PHQ-9 total (0–27)	11.7 ± 7.1	17.4 ± 6.1	20.8 ± 5.8	<0.001
ISI total (0–28)	10.3 ± 5.3	15.7 ± 6.4	18.3 ± 6.2	<0.001
PSS-10 total (0–40)	18.2 ± 9.9	24.1 ± 7.8	27.8 ± 9.1	<0.001
Female sex	19 (59.4%)	23 (69.7%)	22 (68.8%)	0.627
Urban residence	21 (65.6%)	25 (75.8%)	18 (56.2%)	0.252
High scarring (grade ≥ 2)	10 (31.2%)	16 (48.5%)	19 (59.4%)	0.075
High QoL impairment (DLQI ≥ 11)	13 (40.6%)	26 (78.8%)	28 (87.5%)	<0.001
Moderate-to-severe depression (PHQ-9 ≥ 10)	19 (59.4%)	29 (87.9%)	31 (96.9%)	<0.001

Data are mean ± SD or n (%). GAGS, Global Acne Grading System; DLQI, Dermatology Life Quality Index; CADI, Cardiff Acne Disability Index; PHQ-9, Patient Health Questionnaire-9; ISI, Insomnia Severity Index; PSS-10, Perceived Stress Scale-10; SD, standard deviation.

**Table 2:** Comparison of clinical and psychosocial outcomes by scarring burden (high vs. low scarring).

Outcome	Low Scarring (n = 52)	High Scarring (n = 45)	*p* Value
GAGS severity score	21.1 ± 8.1	26.4 ± 7.1	<0.001
DLQI total (0–30)	14.4 ± 7.6	18.2 ± 7.6	0.016
CADI total (0–15)	7.8 ± 3.6	10.1 ± 3.4	0.002
PHQ-9 total (0–27)	13.4 ± 7.4	20.3 ± 4.9	<0.001
ISI total (0–28)	11.4 ± 6.3	18.6 ± 5.3	<0.001
PSS-10 total (0–40)	19.6 ± 9.4	27.7 ± 8.1	<0.001
High QoL impairment (DLQI ≥ 11)	31 (59.6%)	36 (80.0%)	0.052
Moderate-to-severe depression (PHQ-9 ≥ 10)	35 (67.3%)	44 (97.8%)	<0.001

High scarring defined as grade ≥ 2. DLQI, Dermatology Life Quality Index; CADI, Cardiff Acne Disability Index; PHQ-9, Patient Health Questionnaire-9; ISI, Insomnia Severity Index; PSS-10, Perceived Stress Scale-10; SD, standard deviation.

**Table 3:** Spearman correlation matrix among severity, scarring, QoL impairment, and psychosocial measures.

Spearman ρ	GAGS	Scarring Grade	DLQI	CADI	PHQ-9	ISI	PSS-10
GAGS	—	+0.25 *	+0.62 ***	+0.66 ***	+0.55 ***	+0.53 ***	+0.48 ***
Scarring grade	+0.25 *	—	+0.27 **	+0.31 **	+0.44 ***	+0.49 ***	+0.43 ***
DLQI	+0.62 ***	+0.27 **	—	+0.47 ***	+0.59 ***	+0.49 ***	+0.60 ***
CADI	+0.66 ***	+0.31 **	+0.47 ***	—	+0.53 ***	+0.46 ***	+0.42 ***
PHQ-9	+0.55 ***	+0.44 ***	+0.59 ***	+0.53 ***	—	+0.61 ***	+0.63 ***
ISI	+0.53 ***	+0.49 ***	+0.49 ***	+0.46 ***	+0.61 ***	—	+0.67 ***
PSS-10	+0.48 ***	+0.43 ***	+0.60 ***	+0.42 ***	+0.63 ***	+0.67 ***	—

Values are Spearman ρ. * *p* < 0.05; ** *p* < 0.01; *** *p* < 0.001. GAGS, Global Acne Grading System; DLQI, Dermatology Life Quality Index; CADI, Cardiff Acne Disability Index; PHQ-9, Patient Health Questionnaire-9; ISI, Insomnia Severity Index; PSS-10, Perceived Stress Scale-10.

**Table 4:** Multivariable linear regression predicting dermatology-specific QoL impairment (DLQI total).

Predictor	B	95% CI	*p* Value
GAGS	0.35	0.17 to 0.52	<0.001
Scarring grade	−0.48	−1.56 to 0.59	0.374
PHQ-9	0.27	0.05 to 0.50	0.017
ISI	−0.01	−0.25 to 0.24	0.944
PSS-10	0.24	0.07 to 0.41	0.007
Age years	−0.03	−0.31 to 0.24	0.818
Female	2.35	−0.01 to 4.70	0.051

OLS, ordinary least squares; CI, confidence interval; DLQI, Dermatology Life Quality Index; GAGS, Global Acne Grading System; PHQ-9, Patient Health Questionnaire-9; ISI, Insomnia Severity Index; PSS-10, Perceived Stress Scale-10.

**Table 5:** Logistic regression predicting high QoL impairment (DLQI ≥ 11).

Predictor (Standardized)	OR	95% CI (Bootstrap)	*p* Value (Bootstrap)
GAGS	1.97	1.24 to 3.71	<0.001
Scarring grade	0.70	0.34 to 1.34	0.313
PHQ-9	2.10	1.12 to 4.42	0.020
ISI	1.56	0.69 to 3.80	0.253
PSS-10	2.06	1.23 to 4.72	<0.001
Female	1.38	0.75 to 2.49	0.220

OR, odds ratio; CI, confidence interval. Predictors were z-standardized. DLQI, Dermatology Life Quality Index; GAGS, Global Acne Grading System; PHQ-9, Patient Health Questionnaire-9; ISI, Insomnia Severity Index; PSS-10, Perceived Stress Scale-10.

**Table 6:** Unsupervised psychodermatology phenotypes (k-means clustering) and clinical/psychosocial profiles.

Phenotype	GAGS	DLQI	PHQ-9	ISI	PSS-10	DLQI ≥ 11
Cluster 1 (n = 31)	16.1 ± 5.1	8.1 ± 4.1	8.8 ± 5.2	8.3 ± 4.2	14.4 ± 7.6	6 (19.4%)
Cluster 2 (n = 25)	32.4 ± 4.1	23.6 ± 3.9	23.4 ± 3.6	22.2 ± 3.9	32.1 ± 6.8	25 (100.0%)
Cluster 3 (n = 41)	23.8 ± 5.8	17.8 ± 6.2	18.4 ± 4.3	15.2 ± 4.7	24.9 ± 6.6	36 (87.8%)

K-means clustering performed on standardized GAGS, DLQI, PHQ-9, ISI, and PSS-10. GAGS, Global Acne Grading System; DLQI, Dermatology Life Quality Index; PHQ-9, Patient Health Questionnaire-9; ISI, Insomnia Severity Index; PSS-10, Perceived Stress Scale-10.

**Table 7:** Serial mediation (bootstrap) for the pathway scarring → stress → depressive symptoms → QoL impairment.

Effect	Estimate	95% CI	*p*/Method
a1: Scarring → PSS-10 (per +1 grade)	2.72	1.32 to 4.12	<0.001
d: PSS-10 → PHQ-9 (per +5 points)	1.58	—	<0.001
b1: PHQ-9 → DLQI (per +5 points)	1.37	—	<0.001
b2: PSS-10 → DLQI (per +5 points)	1.18	—	0.003
c’: Scarring → DLQI (direct)	−0.49	−1.53 to 0.55	0.349
Indirect (serial): Scarring → PSS → PHQ → DLQI	0.24	0.03 to 0.53	bootstrap
Indirect (PSS only): Scarring → PSS → DLQI	0.65	0.22 to 1.32	bootstrap
Total effect (direct + indirect)	0.38	−0.58 to 1.42	bootstrap

Indirect effects estimated with bootstrap. DLQI, Dermatology Life Quality Index; PHQ-9, Patient Health Questionnaire-9; PSS-10, Perceived Stress Scale-10.

**Table 8:** Internal consistency reliability (Cronbach’s α) of questionnaire instruments.

Instrument	Cronbach’s α
DLQI (10 items)	0.92
CADI (5 items)	0.79
PHQ-9 (9 items)	0.92
ISI (7 items)	0.89
PSS-10 (10 items)	0.92

Cronbach’s α interpreted as internal consistency reliability. DLQI, Dermatology Life Quality Index; CADI, Cardiff Acne Disability Index; PHQ-9, Patient Health Questionnaire-9; ISI, Insomnia Severity Index; PSS-10, Perceived Stress Scale-10.

**Table 9:** Top partial correlation edges from a regularized network model (Graphical Lasso).

Top Partial Correlation Edges	Partial r
ISI—PSS-10	+0.37
GAGS—DLQI	+0.37
PHQ-9—PSS-10	+0.25
DLQI—PSS-10	+0.24
DLQI—PHQ-9	+0.22
Scarring_grade—ISI	+0.21
PHQ-9—ISI	+0.19
Scarring_grade—PHQ-9	+0.17
GAGS—ISI	+0.17
GAGS—PHQ-9	+0.16

Graphical Lasso = regularized inverse covariance model; edges are partial correlations. GAGS, Global Acne Grading System; DLQI, Dermatology Life Quality Index; PHQ-9, Patient Health Questionnaire-9; ISI, Insomnia Severity Index; PSS-10, Perceived Stress Scale-10.

**Table 10:** Discrimination performance (AUC) for predicting high QoL impairment (DLQI ≥11) using 5-fold CV.

Model	AUC (5-Fold CV)
Clinical model (GAGS + scarring + age + sex)	0.780
Psychosocial model (PHQ-9 + ISI + PSS-10 + CADI)	0.848
Combined model (clinical + psychosocial)	0.841

AUC, area under the ROC curve; CV, cross validation; DLQI, Dermatology Life Quality Index.

**Table 11:** Sensitivity analysis: DLQI total across treatment intensity strata, unadjusted and after adjustment for clinician-rated severity and scarring (analysis of covariance).

Model	Topical Only (n = 32)	Oral Antibiotic + Topical (n = 33)	Isotretinoin (n = 32)	*p* Value (Group)
Unadjusted mean DLQI	11.1	16.9	20.4	<0.001

Unadjusted values are the observed group means reported in Table 1. DLQI, Dermatology Life Quality Index; GAGS, Global Acne Grading System.

## Data Availability

The data presented in this study are available on request from the corresponding author.
